# Efficient electromagnetic transducers for spin-wave devices

**DOI:** 10.1038/s41598-021-97627-3

**Published:** 2021-09-15

**Authors:** David A. Connelly, Gyorgy Csaba, Hadrian Renaldo O. Aquino, Gary H. Bernstein, Alexei Orlov, Wolfgang Porod, Jonathan Chisum

**Affiliations:** 1 Department of Electrical Engineering, University of Notre Dame, Notre Dame, IN 46556 USA; 2 Faculty for Information Science and Bionics, Pázmány Péter Catholic University, Prater u. 50/a, 1083 Budapest, Hungary

**Keywords:** Electrical and electronic engineering, Electronic and spintronic devices

## Abstract

This paper presents a system-level efficiency analysis, a rapid design methodology, and a numerical demonstration of efficient sub-micron, spin-wave transducers in a microwave system. Applications such as Boolean spintronics, analog spin-wave-computing, and magnetic microwave circuits are expected to benefit from this analysis and design approach. These applications have the potential to provide a low-power, magnetic paradigm alternative to modern electronic systems, but they have been stymied by a limited understanding of the microwave, system-level design for spin-wave circuits. This paper proposes an end-to-end microwave/spin-wave system model that permits the use of classical microwave network analysis and matching theory towards analyzing and designing efficient transduction systems. This paper further compares magnetostatic-wave transducer theory to electromagnetic simulations and finds close agreement, indicating that the theory, despite simplifying assumptions, is useful for rapid yet accurate transducer design. It further suggests that the theory, when modified to include the exchange interaction, will also be useful to rapidly and accurately design transducers launching magnons at exchange wavelengths. Comparisons are made between microstrip and co-planar waveguide lines, which are expedient, narrowband, and low-efficiency transducers, and grating and meander lines that are capable of high-efficiency and wideband performance. The paper concludes that efficient microwave-to-spin-wave transducers are possible and presents a meander transducer design on YIG capable of launching $\varvec{\lambda = 500}\,$nm spin waves with an efficiency of − 4.45 dB and a 3 dB-bandwidth of 134 MHz.

## Introduction

Spin-wave-based computing, such as Boolean computing and analog wave-based computing, is currently being researched heavily as an ultra-low-power alternative to complementary metal-oxide-semiconductor (CMOS) computing^1–5^. While a complete and purely spin-wave computing system is unlikely to replace charge-based logic with the current state-of-the-art materials^1^, hybrid spin-wave-CMOS systems hold promise for outperforming purely charge-based circuits as suggested by simulation-based benchmarks of the power-delay-area product^6^. Consequently, scalable and efficient transduction that converts information between the charge and spin-wave domains is an unresolved yet critical prerequisite to these hybrid computing systems, not just because of the direct impact to overall system performance but also because the transduction metrics will inform the optimum partitioning within hybrid spin-wave-CMOS circuits^1^.

While various mechanisms exist by which to convert charge to spin-waves and vice versa, such as spin-transfer and spin-orbit torque, magnetoelastic transduction, and voltage controlled magnetic anisotropy, this paper focuses on assessing and designing inductive antennas that couple AC fields to spin waves. Many other applications, not just Boolean computing, can benefit from these types of transducers. Interference-based devices are well suited to implementing certain mathematical operations and algorithms, such as Fourier transforms^7^, prime factorization^8^, parallel data processing^9,10^, and pattern recognition^11^. Moreover, scalable and efficient inductive transducers would enable on-chip microwave components, such as tunable and programmable filters for radio front-ends, true-time delays, signal to noise enhancers (SNEs) and frequency selective limiters (FSLs)^12^.

Each application will likely have different transduction requirements, such as frequency of operation, wavenumber, absolute and percent bandwidth, efficiency, and input-power for linear or non-linear operation. For instance, tunable filters for radio front-ends require wideband capability but may be agnostic to the chosen spin-wave wavenumber, thereby allowing more efficient transduction of large wavelengths. In contrast, Boolean computing requires relatively narrowband operation at nanoscale wavelengths. Analog wave computing requires many wavelengths in a decay length in order to complete a computation, which implies a need for low-damping magnetic films and linear operation of the film (low input-power levels). In contrast, signal-to-noise enhancers (SNEs) and frequency-selective limiters (FSLs) require highly absorptive materials that behave non-linearly when excited by high RF input-powers. Spin waves are capable of very low energies ($$\upmu
$$eV)^1^, and depending on the magnetic film, spin waves can start behaving non-linearly with as little as 10 nW when a 20 nm thick YIG film is excited by a 1 $$\upmu$$m long transducer. Our paper deals with spin waves in the linear regime when the power carrier by them is below this value.

**Figure 1:**
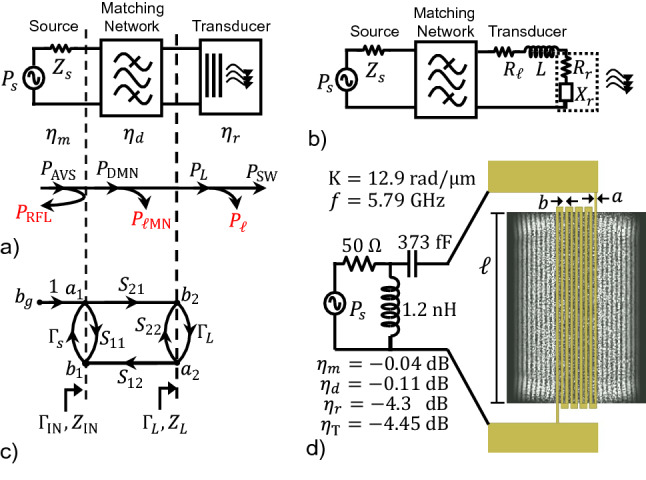
System-level model and example of efficient EM-to-spin-wave transducer. (**a**) Block diagram of the transducer system and the power flow through the system. (**b**) Lumped element approximation to transducer circuit model. The transducer consists of the electromagnetic circuit’s losses ($R_\ell$) and inductance (*L*) and the magnetic film’s radiation resistance ($R_r$) and reactance ($X_r$). (**c**) Signal flow graph for the transducer system. (**d**) Example transducer with matching network and corresponding efficiencies as described in “Efficient, narrowband multi-conductor transducers”.

Currently, spin-wave transduction is inefficient for two reasons. A complete system-level efficiency assessment has not been done^1^, especially for nanoscale transducers, and there is a lack of fully-coupled electromagnetic–micromagnetic simulation tools. Figure 1a presents an end-to-end model that includes a microwave source, impedance matching, and a circuit model of the transducer, thereby enabling a comprehensive system-level analysis that addresses this void in the literature. Microstrip and coplanar waveguide (CPW) transmission lines are commonly used transducers because their cross-sectional mode can be matched to a spin-wave mode while maintaining a characteristic-impedance match to the microwave system. In practice, however, since the transducers are electromagnetically small and the film has a low radiation impedance, the transducer exhibits a large impedance mismatch compared to the RF system. This mismatch results in most of the power being reflected at the input of the transducer. Thus, the subject of impedance matching the spin-wave transducer to the RF source, while largely neglected, must be addressed if efficient energy conversion is to be achieved. This paper demonstrates a practical framework for designing matching networks (MN) of spin-wave transducers that enables efficient, wide-band transduction systems.

The lack of cross-domain simulation tools, which would facilitate the design and study of these transducers, compounds the problem of lossy transduction. Many single-domain simulators exist that model Maxwell’s equations (e.g. Ansys Electronics Desktop HFSS) and micromagnetics (e.g. OOMMF^13^, MuMax^3^^14^), but until recently, no simulator has attempted to fully couple Maxwell’s equations with the Landau–Lifshitz–Gilbert (LLG) equation due to the computationally intensive requirements. In Yao et al.^15^, an implicit finite difference time domain (FDTD) solver couples Maxwell’s equations to the LLG equation but excludes the exchange interaction, which is the dominant interaction for the small wavelengths that wave-based computing applications require to become a competitive alternative to CMOS.

While spin-wave transduction has historically received little attention numerically, it has been studied substantially theoretically in the magnetostatic regime^16–22^. In these works, the radiation resistance is derived assuming a current distribution that is unaffected by the spin waves. Recent publications have numerically coupled the transducer current density to magnetostatic waves by using the method of moments (MoM)^23^ for surface and backward volume waves, analysis of the leaky mode excitation in microstrip lines^24^ for surface waves, and the integral kernel expansion method^25^ for surface and backward volume waves. These works, as well as the previous theoretical derivations, utilize the magnetostatic Polder susceptibility tensor, which is a bulk, linear, and homogeneous approximation of the LLG equation.

While these numerical methods are promising for analyzing transducers in the magnetostatic regime, there is a void of tools focused on the exchange regime. As an alternative way to design exchange-based transducers, one can modify the Polder susceptibility tensor to include the exchange interaction^21,22,26^, thereby extending the theoretical expressions for radiation impedance into the exchange regime. This has several benefits: it allows for rapid design iteration, it preserves the intuitive insight provided by closed-form expressions, and it eliminates the requirement to mesh at increasingly fine spatial scales compared to electromagnetic (EM) wavelengths. In this work, we adopt this approach and compare its benefits and limitations with a numerical approach using the finite element method (FEM) and the magnetostatic Polder susceptibility tensor.

In this paper, we provide a much-needed system-level assessment of inductively-coupled spin-wave transducers, present a computationally-efficient design methodology, and demonstrate examples of practical transducer designs. This methodology can be extended to design efficient transducers in launching waves in the exchange regime. In “Transducer theory”, we review fundamental spin-wave transducer and matching theory and outline the system model utilized in this paper, which is valid for a linear spin-wave regime, typically for RF input-power levels lower than -30 dBm. We also define two figures of merit (FoM): total transducer efficiency and transducer bandwidth. Furthermore, “Transducer theory” discusses the design parameters available to synthesize different transducer responses in k-space and frequency. In “Design methodology”, we demonstrate a theoretically-based, rapid-design approach using a magnetostatic forward volume-wave (MFVW) CPW transducer and validate the approach using an FEM numerical solution (Ansys HFSS), which was first explored by^27^. An optimal matching network is designed for the CPW transducer, resulting in a highly efficient narrow-band transducer.

This systematic design method is then applied, in “Classes of practical transducers”, to various transducer classes based on multi-conductor lines, which are not only capable of achieving wide-band transduction but are also suitable for efficient, nanoscale transducers. The radiation impedance of multi-conductor lines has previously been analyzed^28–31^, but the full benefits of multi-conductor lines, such as wide-band transduction, higher radiation resistance enabling efficient MNs, and efficient transduction at shorter wavelengths have not been fully considered nor appreciated. Conclusions for various applications and recommendations for future work are presented in “Conclusion”.

## Transducer theory

In this section, we first present the system model schematically shown in Fig. 1 and define two important figures of merit (FOM): transducer efficiency ($\eta_{\text{T}}$) and bandwidth (BW). Then we discuss the transducer radiation impedance and finally discuss the theoretical efficiency limits of matching networks.

### System model and figures of merit

The transducer-system efficiency is defined in Eq. (1) as the ratio of spin-wave power ($P_{\text{SW}}$) to the maximum RF power available from the source ($P_{\text{AVS}}$). The maximum power available from the source is defined as the power delivered to the conjugate match, $\Gamma_{\text{IN}}=\Gamma_s^*$. A MN should be included between the transducer and the RF source in order to deliver this maximum power into the spin wave. A illustrated in Fig. 1, power originates from the RF source ($P_{\text{AVS}}$) and can either be reflected ($P_{\text{RFL}}$) or be delivered into the MN ($P_{\text{DMN}}$). Some of $P_{\text{DMN}}$ is dissipated (lost) in the network ($P_{\ell \text{MN}}$) while the rest is transferred to the transducer (load) (*P*_L_). Some *P*_L_ is not delivered into the film in the form of the intended spin-wave mode ($P_{\text{SW}}$) but is dissipated in ohmic or dielectric losses or even higher-order spin-wave modes ($P_{\ell }$).

The system’s total efficiency is the product of three efficiencies: match efficiency ($\eta_{m}$), dissipation efficiency in the MN ($\eta_{d}$), and radiation efficiency of the transducer ($\eta_{r}$). Each efficiency is defined by a power ratio describing the power flow throughout the system (Fig. 1a):

1 $$\begin{aligned} \eta_\text{T} = \frac{P_{\text{SW}}}{P_{\text{AVS}}} = \frac{P_{\text{DMN}}}{P_{\text{AVS}}}\frac{P_{\text{L}}}{P_{\text{DMN}}}\frac{P_{\text{SW}}}{P_{\text{L}}} = \eta_{m}\eta_{d}\eta_{r}. \end{aligned}$$

The match efficiency is given by

2 $$\begin{aligned} \eta_{m} = \frac{P_{\text{DMN}}}{P_{\text{AVS}}} = \frac{\big (1-|\Gamma_{s}|^2\big )\big (1-|\Gamma_{\text{IN}}|^2\big )}{|1-\Gamma_s\Gamma_{\text{IN}}|^2}, \,\,\text{where}\,\, \Gamma_{\text{IN}} = S_{11}+\frac{S_{12}S_{21}\Gamma_{L}}{1-S_{22}\Gamma_{L}}, \end{aligned}$$

where $\Gamma_{s}$ is the reflection coefficient of the source and $\Gamma_{\text{IN}}$ is the reflection coefficient seen looking into the MN terminated by the load $\Gamma_L$ (Fig. 1b). Equation (2) is general for when the RF source has a characteristic impedance other than 50 $\Omega$ (the typical RF-system impedance), which may be the case if the source is a wideband antenna; Eq. (2) reduces to $1-|\Gamma_{\text{IN}}|^2$ when $Z_s = 50$. The first term in $\Gamma_{\text{IN}}$, $S_{11}$, accounts for the power reflected at the first reference plane (between source and MN), and the second term accounts for the portion of the power reflected at the second reference plane (between MN and load) that makes it back to the source (after undergoing multiple reflections).

The dissipation efficiency of the MN is given by^32^

3 $$\begin{aligned} \eta_{d} = \frac{P_{\text{L}}}{P_{\text{DMN}}} = \frac{1}{1-|\Gamma_{\text{IN}}|^2}|S_{21}|^2\frac{1-|\Gamma_L|^2}{|1-S_{22}\Gamma_L|^2}. \end{aligned}$$

The power dissipated in the MN is not only a function of the network but also depends on the network’s termination. This is further discussed in “Matching considerations”, and the practical considerations for implementing a MN are discussed in “Design methodology”.

The radiation efficiency is defined as

4 $$\begin{aligned} \eta_{r} = \frac{P_{\text{SW}}}{P_{\text{AVMN}}} = \frac{R_r}{R_r+R_{\ell }}, \end{aligned}$$

where $R_r$ is the radiation resistance representing real power transferred from the transducer into the magnetic film, and $R_\ell$ is the loss resistance representing all other mechanisms of power lost in the transducer. These mechanisms include ohmic and substrate losses, and for volume waves, also include power lost through higher-order thickness modes. In this paper, substrate conduction loss is ignored for clarity, while the magnetic film’s conduction loss and higher-order thickness modes are negligible since thin YIG films are utilized.

To efficiently launch spin waves, transducers must be geometrically on the order of a micromagnetic wavelength, which means they are small electromagnetically and, therefore, can be modeled by frequency-dependent lumped elements. Figure 1b provides a commonly used model for a transducer consisting of a series ohmic loss ($R_{\ell }$) and line inductance (*L*) in series with a radiation impedance presented by the magnetic film (shown in dashed box). A transducer is best terminated in a short to provide the strongest current and highest radiation efficiency. More importantly, if it were terminated in a 50-ohm port, a greater fraction of the power available from the network would be dissipated in the port impedance rather than in the film. It is important to note that while the transducer’s electrodes are physically shorted, the transducer adjacent to a magnetic film is not electrically shorted because the film absorbs power. Therefore, when matching to a transducer, one is matching to the radiation impedance due to the magnetic film (in addition to any losses and reactances of the line itself), not to a short circuit.

The second important figure of merit is bandwidth (BW) because both the radiation and match efficiency vary with frequency. Bandwidth may need to be defined differently for each application, but a general rule that we will use here is the band within which the $\eta_{\text{T}}$ drops by no more than 3 dB from the maximum efficiency. Bandwidth is inversely proportional to match efficiency^33,34^; it is easy to match well at a single frequency but becomes increasingly difficult to match over a wide band.

### Calculation of the radiation impedance

Since the design of matching networks and simulation of transducer currents can be easily achieved with commercial solvers such as Keysight ADS and Ansys HFSS, the challenge lies in accurately modeling the load impedance that spin waves place on the transducer. Several theoretical treatments have been done for magnetostatic forward waves^20–22^, for backward volume waves^19^, and for surface waves^17^. These approaches start from the definition of radiation impedance, which relates the transducer current to the excited spin-wave power, and then calculate the excited spin-wave power using the magnetostatic Poynting vector and a 3D overlap integral of the spin-wave and source magnetic potential functions.

The definition of radiation impedance $Z_r = R_r +jX_r$ is given by relating the power in an excited spin wave, $P_{\text{SW}}$, to the applied current, $I_a$, that excited the spin wave:

5 $$\begin{aligned} P_{\text{SW}} = \frac{1}{2}Z_r|I_a|^2 = \frac{1}{2}\big (R_r+jX_r\big )|I_a|^2. \end{aligned}$$

For a theoretical framework to calculating the power in the excited magnetostatic wave, see^1,17,21,22^.

In all cases, closed-form expressions for the radiation resistance are derived theoretically, and the reactance is obtained numerically by applying Kramers–Kronig relations^18^. We restrict our focus to MFVW radiation resistance in this work because the propagation of volume waves is independent of any direction normal to the bias field and thus are inherently well-suited for wave-based computing devices. It is worth noting that magnetostatic backward volume waves^19^ (MBVW) have a radiation resistance of similar magnitude as MFVW, while surface waves^17^ have an order of magnitude higher $R_r$. The radiation resistance for the *n*th thickness mode ($n = 0, 2, 4$...for even, $n = 1, 3, 5$...for odd) is^20–22^

6 $$\begin{aligned} R_{r,n} = \frac{\ell \omega \mu_o\cos ^2\big [\frac{k_n\,d}{2}\sqrt{-(1+\chi_d)}-\frac{n\pi }{2}\big ]}{[-(1+\chi_d)]k_n\,d}\big |{\mathscr {J}}_{sFT}\big |^2 = r_i\big |{\mathscr {J}}_{sFT}\big |^2, \end{aligned}$$

where $\ell$ is the transducer length, $\omega$ is angular frequency, $\mu_o$ is the permeability of free space, $\chi_d$ is the diagonal component of the Polder susceptibility tensor^21,22,35^ (see section Supplemental S2 for more), $k_n$ is the wavenumber for the nth mode (see section Supplemental S4 for a discussion on the dispersion relation), *d* is the film thickness, $r_i$ is the intrinsic radiation resistance described further below, and ${\mathscr {J}}_{sFT}$ is the spatial Fourier transform (FT) of the cross-section of the surface current density normalized by the applied current, $I_a$.

The radiation resistance of a spin wave can be separated into three distinct components:

7 $$\begin{aligned} R_r = r_i r_{sf} = r_i r_{ef} r_{af}, \end{aligned}$$

where the surface current factor $r_{sf} = |{\mathscr {J}}_{sFT}|^2$, can be written as the product of an element factor, $r_{ef}$, and an array factor, $r_{af}$. In general, $r_{sf}$ is a function of wavenumber (and frequency). The intrinsic radiation resistance, $r_i$, is the resistance seen looking into an infinitesimal current filament that launches spin waves, and is a function of the film thickness, saturation magnetization, $M_s$, bias field strength, and dispersion relation. The intrinsic radiation resistance can be understood as the line-spread function of the magnetic system when excited by a current filament. The current filament acts as a spatial impulse source having a ${\mathscr {J}}_{sFT}(k) = 1$, and the $r_i$ is the impulse response of the magnetic system. Hence, since the intrinsic radiation resistance is defined with respect to a single current filament, it is independent of the transducer geometry and is the resistance limit of a single-conductor transducer.

A transducer with a high intrinsic radiation resistance (near 50 $\Omega$) is desirable in order to maximize $\eta_\text{T}$, but some applications do not allow independent tuning of the parameters comprising $r_i$. One might be tempted to simply scale the transducer length, $\ell$, since the radiation resistance scales directly with $\ell$, but long transducers ($\ell \,\ge$ 1 mm) are difficult to implement as integrated circuits. As a result, designers must consider which of the parameters that comprise $r_i$ are available to increase $r_i$. After $r_i$ has been maximized given specific device requirements, the transducer geometry must be designed to provide the appropriate $r_{sf}$ to achieve the required total radiation resistance, $R_r$, over the desired wavenumber (and frequency) of operation.

For instance, the bias field and saturation magnetization set the FMR frequency (cutoff frequency) of the transducer. Given practical constraints on physical transducer size (fabrication limitations or system efficiency requirements), which set the maximum wavenumber, the saturation magnetization, also sets the maximum operating frequency and, consequently, the absolute bandwidth. However, in the case of wave-based computing applications, materials are chosen primarily based on their saturation magnetization and damping factor in order to achieve the longest decay length per wavelength^1^. The bias field is then set according to the desired band of operation, with a stronger bias yielding a higher $r_i$. The film thickness should be selected based on the type of spin-wave. For volume spin waves, thinner films yield higher radiation resistances than thicker films because they suppress higher order thickness modes, whereas for surface spin waves, thicker films yield a higher radiation resistance.

While current varies longitudinally along a transmission line on the order of an electromagnetic wavelength, the cross-section of the line can be reduced so it is on the order of a micromagnetic wavelength. The key is matching the cross-sectional mode(s) of the current distribution to the spin-wave mode of interest. To this end, synthesis of the surface current factor, $r_{sf}=r_{ef}r_{af}$, is the primary means of obtaining the desired wavenumber (and frequency) response. The element factor, $r_{ef}$, represents the Fourier sum of current filaments that comprise an individual conductor; this distribution results from the skin effect and the proximity effect, which typically cannot be engineered since they are dictated by Maxwell’s equations. The array factor, $r_{af}$, on the other hand, can be engineered; it represents the Fourier sum of multiple conductors after the common distribution, $r_{ef}$, has been factored out. The array factor provides significant freedom in synthesizing frequency selection and enables the increase of $R_r$ beyond the intrinsic limit. Therefore, the primary means of synthesizing $R_r(k)$ is to engineer the spacing, size, and number of the conductors. “Classes of practical transducers” demonstrates simulated designs, and section Supplemental 1 provides additional theory behind the synthesis of $r_r(k)$.

### Matching considerations

In general, a spin-wave transducer does not have uniform nor source-matched impedance over a wide band of frequencies. Short volume-wave transducers ($\ll$ 100 $\upmu$m) typically exhibit a significant impedance mismatch with the RF source, resulting in very low power delivered to the transducer if no matching network is used. Therefore, any efficient transducer design must include a MN and must consider not only the quality of the match ($\eta_m$) but also the dissipation loss of the match ($\eta_d$). This is especially true because loss in a MN increases as the impedance mismatch increases. While Eq. (3) provides the exact power dissipated if the MN and load s-parameters are known, it does not provide much intuition.

Consider, for instance, an L-section MN comprised of a lossy inductor and lossy capacitor of equal element quality factor $Q_e$. If the element quality factor is much higher than the overall quality factor of the network, $Q_n$, then the dissipation loss (Eq. 3) can be approximated as follows^36,37^:

8 $$\begin{aligned} \eta_d = \frac{P_L}{P_{\text{DMN}}} \approx \frac{1}{{\big (}1+\frac{Q_n}{Q_e}{\big )^2}} = \frac{1}{{\big (}1+\frac{\sqrt{\text{ITR}-1}}{Q_e}{\big )^2}}. \end{aligned}$$

The impedance transformation ratio (ITR) is defined as^37^

9 $$\begin{aligned} \text{ITR} = \frac{\mathfrak {R}\{Z_{\text{IN}}\}}{R_L} = Q_n^2+1, \end{aligned}$$

where $R_{L} = R_r+R_{\ell }$ is the real part of the transducer impedance and $\mathfrak {R}\{Z_{\text{IN}}\}$ is the real part of the input impedance seen from the reference plane between the source and MN (Fig. 1c). The first significance of Eq. (8) is that the smaller the transducer’s resistance, $R_L$, compared to the source resistance, $Z_s$, the larger the ITR requirement on the MN, and the greater the dissipation loss of the network for a fixed element quality-factor, $Q_e$. Therefore, there is a trade-off between a network that provides the best match (Eq. 2), which would require a higher order (higher complexity) network, and a network that has low dissipation loss, which would require a lower order (lower-complexity) design. The second significance of Eq. (8) is that a transducer design with higher radiation resistance, such as meander lines discussed in “Efficient, narrowband multi-conductor transducers”, allows for a more efficient MN resulting from a lower ITR requirement. While Eq. (8) holds true for any MN design, the very low $R_r$ of MFVW transducers makes the relationship between $\eta_d$, ITR, and $Q_e$ especially pronounced, as is further discussed in “Efficient, narrowband multi-conductor transducers” and illustrated by Fig. 6d.

Equation (8) is not limited to a lumped-component L-section but also applies to a distributed network; in fact, all the physically realized networks designed for narrowband transducers studied in “Design methodology” and “Classes of Practical Transducers” and described in Table 1 are distributed L-sections. Despite being the simplest network topology, L-sections provide good matching across the band while minimizing dissipation losses that would result from higher-order networks. Wideband transducers in “Efficient, wideband multi-conductor transducers” require more-sophisticated matching methods, such as the real frequency technique (RFT)^38–40^, in order to address the frequency-dependent load impedance.

**Table 1:** CPW matching network parameters and dimensions.

Transducer		$\hbox {TX}_1$			$\hbox {TX}_2$		
*a*	$\ell$	$Z_o$	$\beta l$	W / G / L	$Z_o$	$\beta l$	W / G / L
($\upmu$m)	($\upmu$m)	$\Omega$	($^\circ$ SC)	(mm)	$\Omega$	($^\circ$)	(mm)
1	100	41.5	29.1	1 / 0.154 / 2	104	168	1 / 1.74 / 14.62
1	10	49.8	7.1	1 / 0.274 / 0.5	45.0	169	1 / 0.201 / 11.72
2	10	45.2	7.2	1 / 0.203 / 0.5	42.6	169	1 / 0.168 / 11.69
3	10	44.0	7.2	1 / 0.186 / 0.5	40.5	169	1 / 0.141 / 11.58
5	10	42.6	7.2	1 / 0.167 / 0.5	41.5	169	1 / 0.153 / 11.65

## Design methodology

In this section, we present a method for the systematic design of a transducer as outlined in steps 1–7 below. The method is based on theoretical, closed-form expressions for the dispersion relation and radiation impedance, and requires an EM simulation of the surface current distribution without modeling any magnetic film. This method is confirmed by a second approach that performs an EM simulation of the transducer launching magnetostatic waves in a magnetic film. While both approaches solve for the radiation impedance of magnetostatic waves to provide an equal comparison here, the theoretical approach can be easily modified to include the exchange interaction, rendering it a computationally efficient approach to nanoscale transducer design. Both these approaches are demonstrated with a CPW transducer, and in “Classes of practical transducers”, we show additional examples using the same procedures.

**Figure 2:**
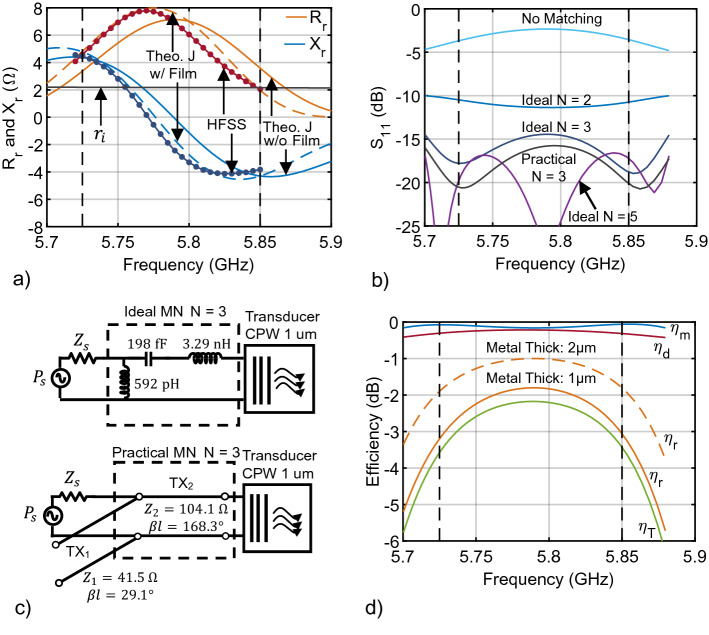
Summary of system performance for a CPW transducer targeting the LTE-U band. (**a**) Radiation impedance for a 100 $\upmu$m long CPW transducer (a = 1 $\upmu$m) targeting the LTE-U band. The out-of-plane applied bias field is 0.3763 T. Solid orange and blue curves are the radiation resistance ($R_r$) and reactance ($X_r$), respectively, obtained from the theoretical approach. Markered red and dark blue lines are the radiation impedance obtained from the second approach. Dashed orange and blue lines represent with theoretical radiation impedance computed by modifying step 3 of the theoretical approach. (**b**) Simulated $\Gamma_{\text{L}}$ and $\Gamma_{\text{IN}}$ with lumped-component MNs of various orders. (**c**) Lumped circuit-model of $N = 3$ MN and its physical realization using a CPW short-circuited stub and line. Network dimensions are given in Table 1. (**d**) The radiation efficiency ($\eta_r$) for two CPW metal thicknesses, match efficiency ($\eta_m$) and dissipation efficiency ($\eta_d$) using the physical CPW network in (**c**), and overall transducer efficiency ($\eta_\text{T}$).

To design an efficient transducer, the following theoretical methodology is employed:

1. The cross-sectional size of the transducer is set to be on the order of the magnon wavelengths (frequencies) of interest.
2. An EM simulation of the transducer is performed without a magnetic film to determine the surface current distribution.
3. A cross-sectional line-cut of the current distribution is exported to compute ${\mathscr {J}}_{sFT}$.
4. The theoretical dispersion relation (Eq. 4.75a in^21,22^) is solved numerically for the modes of interest.
5. The radiation resistance is then computed using Eq. (6).
6. The reactance is computed using Kramers–Kronig relations^18^.
7. An appropriate matching network is designed by first obtaining a lumped-element network using the RFT and subsequently applying Richard’s transformations^41^ to convert the lumped circuit to distributed lines and stubs. These lines are modeled with physical dimensions and loss and optimized to achieve the highest $\eta_m\eta_d$ product.

Note that no micromagnetic simulations are needed.

**Figure 3:**
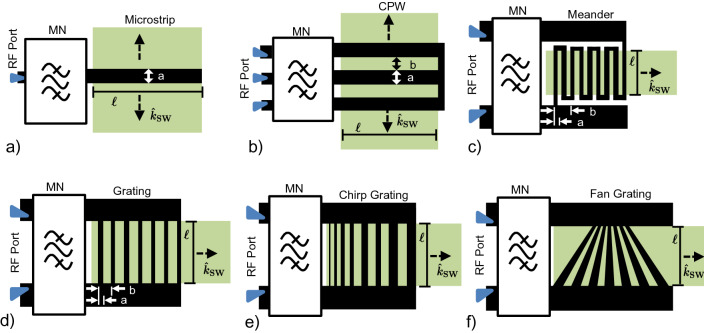
Various classes of transducers. (**a**) Narrow-band single-conductor transducer (microstrip); (**b**) narrow-band multi-conductor transducer (CPW); (**c**,**d**) multi-conductor transmission-line transducers (meander and grating, respectively); (**e**,**f**) wide-band multi-conductor transducers for chirp and fan gratings, respectively.

We now proceed to demonstrate the design of a CPW transducer targeting the Long-Term Evolution Unlicensed (LTE-U) band from 5.725 to 5.85 GHz^42^ so that it can be combined with a chip-scale spectrum analyzer^7^ to enable Listen-Before-Talk^42^. The transducer layout is similar to that depicted in Fig. 3b.

To perform step 1, the fundamental wavelength that a CPW line targets (when $a = b$) can be estimated by 4*a*. A thin YIG film ($d = 100$ nm) is chosen to suppress higher-order thickness modes, and the bias is set to $\hbox {B}_a = 0.3763$ T to center the CPW’s response in the 5.8 GHz band. Hence, the CPW dimension $a = 1\,\upmu$m is selected as the largest dimension that still covers 5.725–5.85 GHz. The transducer’s length was $\ell = 100\,\upmu$m.

**Figure 4:**
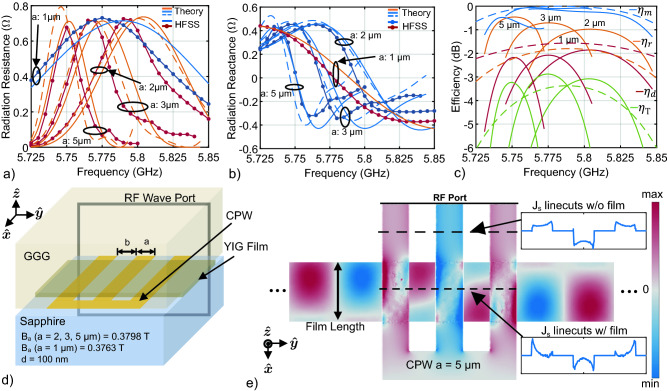
Summary of system performance for various short CPW transducers targeting the LTE-U band. (**a**,**b**) Radiation impedance for several CPW transducers of length 10 $\upmu$m and sizes a = 1, 2, 3, and 5 $\upmu$m covering part of the LTE-U band. Solid orange and blue curves are computed using the theoretical approach outlined in “Design methodology”. Markered red and dark blue lines are the radiation impedance obtained by the numerical approach outlined in “Design methodology”. Dashed orange and blue lines are computed by a modification of step 3 in the theoretical approach. The a = 1 $\upmu$m transducer is biased at B_a_ $= 0.3763$ T while the other transducers are biased at B_a_ $= 0.3798$ T. (**c**) The radiation efficiency ($\eta_r$), match efficiency ($\eta_m$) and dissipation efficiency ($\eta_d$), and total transducer efficiency ($\eta_\text{T}$). Dashed curves represent $a = 1\,\upmu$m efficiencies. Network dimensions are given in Table 1. (**d**) FEM simulation setup of a YIG-on-GGG flip-chipped onto a CPW transducer with a sapphire substrate. (**e**) Top view of the CPW J_s_ and spin wave amplitude H_x_. Insets are 1D line-cuts of J_s_.

Step 2 is accomplished by modeling the CPW structure using Ansys HFSS. This required a fine mesh on the conductors to accurately capture the transverse spatial variation of the current. The EM simulation yields 4.2 $\upmu$m as the fundamental wavelength of the surface current density, which is close to 4*a*. For more details on the EM simulation, see section Supplemental 2. A cross-sectional line-cut of the current density is exported, and the spatial fast Fourier transform (FFT) of this current is computed to obtain ${\mathscr {J}}_{sFT}$ (step 3). The dispersion relation for the fundamental thickness mode is calculated (step 4, see Fig. 4 section Supplemental S4), and the resulting theoretical radiation impedance (steps 5 and 6) is shown in Fig. 2a (solid orange and blue curves).

The reflection coefficient of the transducer, obtained by combining the theoretical radiation impedance with the ohmic losses $R_{\ell }=3.71\,\Omega$ and inductance $L = 38\,$pH of the CPW line, without a MN is shown in Fig. 2b, indicating significant mismatch and low match efficiency at 5.8 GHz. Therefore, a matching network (step 7) is needed to deliver power to this transducer. Because this transducer’s radiation resistance dominates the losses, the transducer’s input impedance varies over frequency in a manner not exactly described by an analytical transfer function; therefore, a broadband matching algorithm, such as the RFT, would benefit this transducer. The well-established RFT^38–40^ is a computer aided design (CAD) procedure that takes frequency-dependent, complex load impedances and optimizes a lossless network’s match efficiency, $\eta_m$, over a specified band without *a priori* assumptions of an analytic network transfer function (e.g. Chebyshev polynomials) or topology. The RFT accepts as input the load’s frequency-dependent s-parameters. The RFT is computationally more efficient and yields simpler and more optimal MNs (for complex source and load impedances) than analytic gain-bandwidth synthesis techniques^43,44^.

Lossless matching networks of several orders (N = 2, 3, and 5) were designed using the RFT and are also shown in Fig. 2b. Generally, a higher-order network provides a better match, although network orders higher than 5 do not produce a better match for this transducer. It is worth noting that even a low-order network ($N\!=\!2$) significantly improves the match efficiency ($\eta_m = 0.9$). In fact, it is preferable to use low-order MNs because, when realized with transmission lines or lossy lumped components, larger complex networks incur more ohmic losses, thereby reducing the dissipation efficiency. Hence, instead of the fifth-order network, a nearly-equal performance third-order network is realized with lossless lumped elements shown in Fig. 2c with a series 3.29 nH inductor, series 198 fF capacitor and shunt 592 pH inductor. Then, using Richard’s transformation^41^, the network is converted to a lossless transmission-line design as shown at the bottom of Fig. 2c. This transmission line network is then converted to a physical CPW stub and line, yielding the practical $N = 3$ curve in Fig. 2b (electrical length defined at 5.8 GHz). A sapphire substrate 500 $\upmu$m thick ($\epsilon_r = 9.3,\, \text {tan}\,\delta = 0.0001$) and 1 $\upmu$m metal thickness were used for the network. The dimensions of this stub and line are found in the first row of Table 1. The resulting match and dissipation efficiency are shown in Fig. 2d (solid curves). Since the radiation efficiency, $\eta_r$, for a transducer metallization thicknesses of 1 $\upmu$m, dominates the match and dissipation efficiency, $\eta_r$ can be improved using thicker metallization (see thickness 2 $\upmu$m dashed orange curve). For transducers launching exchange spin waves, fabrication methods capable of producing high aspect-ratios, such as $19\!:\!1$ with SAFIER-complemented EBL^45^ and greater than $100\!:\!1$ with LIGA^46^, are likely required to maintain a high $\eta_r$.

To validate the theoretical approach, this CPW transducer and YIG film were modeled in HFSS by defining the magnetostatic Polder susceptibility tensor for the YIG film and a uniform bias field of $\hbox {B}_a = 0.3763$ T to target the LTE-U band. This required not only a fine mesh in the conductors but also in the entire film region, which increased the simulation time by an order of magnitude. An absorbing boundary layer (ABL) is created to emulate an infinite film, thereby preventing reflections from returning to the transducer. For more details on the ABL and the simulation setup, see section Supplemental S2. The radiation impedance was extracted by de-embedding the transmission line’s ohmic loss, $R_{\ell }$, and inductance, *L*. The input impedance, $Z_{\textrm{IN}} = 50(1+S_{11})/(1-S_{11})$, of the transducer without a film was subtracted from the input impedance of the transducer with a film using the simulated scattering parameters ($S_{11}$). Since the transducer is electromagnetically a very short line, it can be treated as a lumped series $R_\ell$ and *L* as shown in Fig. 1b. The de-embedded radiation impedance is plotted in Fig. 2a as the dark blue and red curves with markers. While there is close agreement with the theoretical approach, there is a noticeable frequency shift to the left, which is attributed to the spin waves changing the current distribution. To verify this, step 3 of the theoretical approach was modified by using the HFSS current distribution solved with a film present. The result is plotted using dashed lines in Fig. 2a, and is in much better agreement with the theory, indicating that the wavenumbers of the current’s cross-section have changed as a result of the spin waves. This spin-wave-to-current coupling is further discussed in “Multi-conductor transmission-line transducers” and illustrated in Fig. 4e for a larger CPW design.

Despite the simplifying assumption that the current distribution does not change due to the spin waves, the theoretical approach (which uses the much more efficient EM simulation without a film present) still yields fairly accurate results. This means that the theoretical approach is useful for obtaining initial designs, rapidly iterating on a design, designing transducers in the exchange regime, and designing more complex transducers due to a much lower EM-simulator mesh requirement. Nonetheless, an EM simulation with the film is useful for design validation when high accuracy is needed before device fabrication.

## Classes of practical transducers

Using the design methodology and numerical simulations presented above, this section discusses and demonstrates the various classes of transducers that can be designed with efficiency and bandwidth in mind. In general, four classes exist: single-conductor transducers (e.g. microstrip, loop), multi-conductor transmission-line transducers (e.g. CPW), efficient, narrowband multi-conductor transducers, and efficient, wideband multi-conductor transducers. We will show that the first two classes, while often employed and simple to design, result in low efficiencies. This is because a transmission line is inherently suited to guiding electromagnetic energy longitudinally rather than leaking energy transversely into a magnetic film. Therefore, we also present and compare two efficient, narrowband designs and conclude with a discussion on potential wideband, efficient transducers.

### Single-conductor transducers

Figure 3a illustrates a microstrip line as a typical example of a single-conductor transducer. This class of transducers involves minimal design since the current distribution of an individual conductor cannot be synthesized but is set by the skin effect, proximity effect, and the electromagnetic modes that the transmission line can support. Single-conductor transducers act as low-pass structures in k-space: a physically-wide conductor has a low wavenumber cutoff whereas a physically-narrow conductor has a high wavenumber cutoff. This is the extent of their design.

The intrinsic radiation resistance ($r_i$ from Eq. 6) is the upper resistance-limit for a single-conductor transducer; due to a single conductor’s low-pass response, this resistance can be obtained only at the lowest wavenumbers. Unless the transducers are long ($\ell \ge$ mm), the intrinsic radiation resistance remains significantly lower than the RF system impedance, rendering efficient matching difficult for integrated circuits at microwave frequencies. As the conductor becomes physically narrower, the radiation efficiency of single-conductor transducers decreases due to increasing ohmic losses. For this reason, along with their low-pass response, these transducers are unsuitable for efficient excitation of exchange spin waves.

Consequently, single-conductor transducers have limited use and should be avoided in favor of multi-conductor transducers, which can reduce ohmic losses and increase radiation efficiency.

### Multi-conductor transmission-line transducers

The second class of transducers, which includes CPW and co-planar strip (CPS) lines, exhibits better performance than single-conductor transducers, but is neither wideband nor exceptionally efficient. These transducers can still be designed easily, which has made them ubiquitous for launching spin waves. Figure 3b illustrates a CPW whose dimensions *a* and *b* can be engineered to achieve a specific narrow-band response in k-space. This transducer has a bandpass response in k-space; it can be used to excite specific wavenumbers more efficiently than single-conductor transducers but still faces decreasing radiation efficiency with increasing wavenumber. However, the radiation resistance achievable by a CPW (or a CPS) is significantly higher than the intrinsic radiation resistance because $r_{af} > 1$, so it can maintain a given efficiency out to shorter wavelengths as compared to single-conductor transducers, where $r_{af} = 1$.

To demonstrate these characteristics for MFVW, several CPW transducers targeting the LTE-U band were modeled using the theoretical and EM approaches outlined in “Design methodology”. These transducers have a length of 10 $\upmu$m, whereas the transducer from “Design methodology” was 100 $\upmu$m. Figure 4d illustrates the simulation setup, where a film on a GGG substrate is modeled as if it were flip-chipped onto a CPW-patterned sapphire substrate. See section Supplemental S4 for details of the dispersion relation for these transducers. The radiation resistance, reactance and all efficiencies are presented in Fig. 4a–c, respectively. Three CPW geometries ($a\! =\! b \! = 2, 3, \,\text{and}\,5\,\upmu$m) with a bias field of 0.3798 T cover a significant portion of the band, whereas a single $a \!=\! b\! =\! 1\,\upmu$m transducer biased slightly differently (0.3763 T) can cover the entire band, but with lower radiation efficiency.

In general, the theoretical approach (solid, no markers) shows good agreement with the electromagnetic simulation (solid with markers). The slight frequency shift between the theoretically and numerically computed radiation impedance can be partially explained by the change in current distribution resulting from the presence of spin waves as illustrated by Fig. 4e. The inset provides the current distribution of the CPW line at the two indicated linecuts. The current distribution over the film changes and the amplitude peaks are spread further out spatially, resulting in a lower fundamental wavenumber and lower frequency. Even though the current is not uniformly distributed along the length of the CPW line with the film present, assuming a uniform current distribution, as in the case of the theoretical approach, yields a very close result.

All efficiencies are shown in Fig. 4c, where the solid lines represent CPW sizes $a = 2, 3, 5\,\upmu$m biased at B_a_ $= 0.3798$ T and the dashed lines represent the $a = 1\,\upmu$m transducer biased at B_a_ $= 0.3763$ T. In all cases, the match efficiency, $\eta_m$, is very good; it remains above − 1.3 dB for the $a = 2, 3, 5\,\upmu$m lines and above − 0.73 dB for the $a = 1\,\upmu$m line. The peak radiation efficiencies, $\eta_r = -0.46, -0.71, -0.98, -1.8\,$ dB, decreases with decreasing CPW size for $a = 5, 3, 2, 1\,\upmu$m, respectively, which is expected due to increased ohmic losses. The peak dissipation efficiencies, $\eta_d = -2.18, -2.1, -1.86, -1.57\,$ dB, are fairly similar across all devices for $a = 5, 3, 2, 1\,\upmu$m, respectively. The peak total efficiency, $\eta_\text{T} = -3.37\,$ dB, for the $a = 1\,\upmu$m CPW (dashed traces) is lower across this LTE-U band than if it were covered by multiple, larger transducers ($\eta_\text{T} = -3.23, -2.88, -3.08\,$ dB for $a = 5, 3, 2\,\upmu$m, respectively), but it is advantageous in requiring only one device and one MN.

### Efficient, narrowband multi-conductor transducers

As mentioned in “Calculation of the radiation impedance”, the element factor, $r_{ef}$, cannot be synthesized; on the other hand, the array factor, $r_{af}$, can be synthesized in much the same way as antenna elements in a phased array. Therefore, this class of transducers can achieve high radiation and match efficiencies over narrow bandwidths, even at nanoscale wavelengths. This section explores the efficiency of gratings and meander lines (Fig. 3c,d) using the theoretical and numerical methodology outlined in “Design methodology”. See section Supplemental S4 for details of the dispersion relation for these transducers.

**Figure 5:**
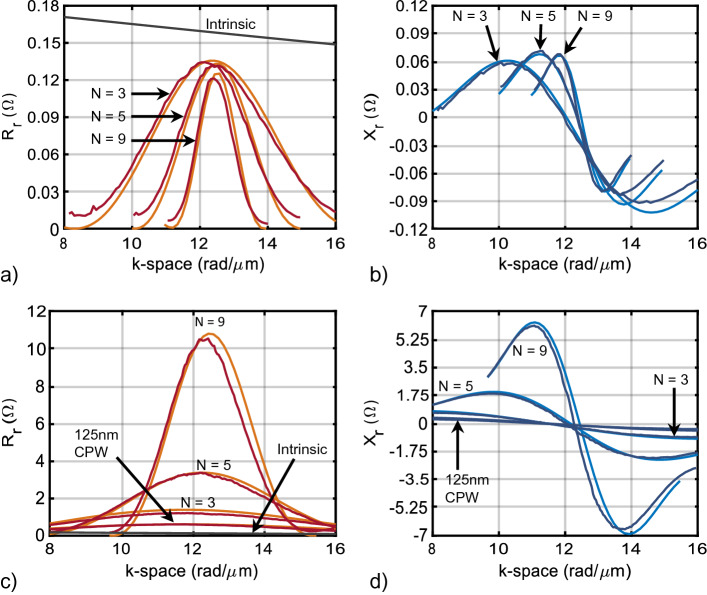
Comparison of the theoretical and FEM radiation impedance for gratings and meander lines as shown in Fig. 3. (**a**,**b**) Radiation resistance and reactance, respectively, for gratings with various number of elements *N*. (**c**,**d**) Radiation resistance and reactance, respectively, for meander lines with various number of elements *N*. Orange and light blue are the impedances obtained with the theoretical approach, while the dark red and blue were obtained using the numerical approach. Film thickness is 100 nm. A uniform bias of 277 kA/m is applied out-of-plane. The length of the transducers is 10 $\upmu$m. The transducers target $\lambda = 0.5\,\upmu$m with $a = 0.125\,$ nm and $b = 0.5\,$ nm. A CPW line of $a = 0.125\,$ nm was also simulated for comparison. The intrinsic resistance is shown for comparison (black).

**Figure 6:**
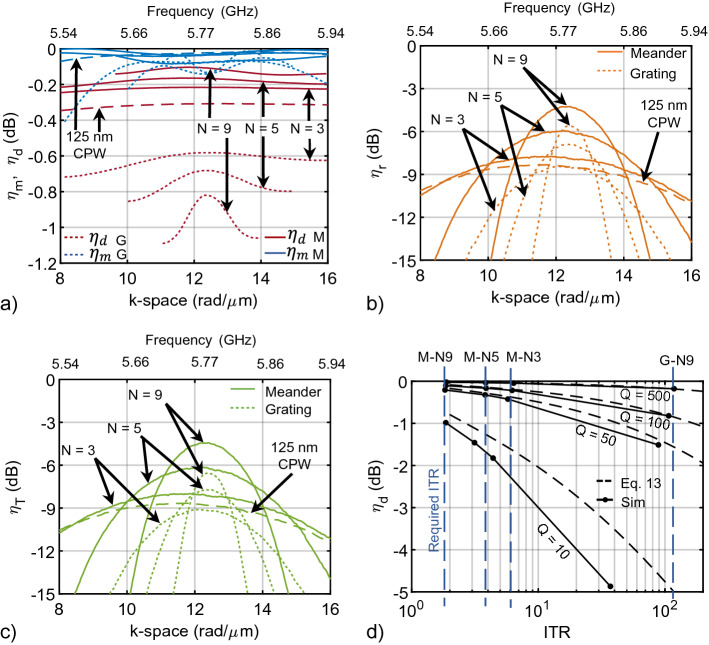
(**a**) The match efficiency for L-section, lumped-element, MNs ($Q_e = 100$) designed for meander (solid), grating (dotted), and CPW (dashed) transducers are shown in blue, whereas the dissipation efficiency of each network is shown in red. (**b**) plots radiation efficiency (Eq. 4 for meander (solid), grating (dotted), and CPW (dashed) transducers simulated in HFSS. The total efficiency (Eq. 1) is shown in (**c**) for meander (solid), grating (dotted), and CPW (dashed) transducers. (**d**) Dissipation efficiency as a function of ITR for MNs of constant quality factor $Q_e = 10,\,50,\,100, \,\,\text{and}\,\,500$. The approximation in Eq. (8) is plotted as a dashed, black line. The solid black line with markers represents the dissipation efficiency at the ITR achieved with the finite-Q networks for a grating with N = 9, and meanders with N = 3, 5, and 9. The required ITR for each device to achieve a perfect match is shown by the dashed blue lines, but as a result of adding finite Q after designing the networks, they do not provide a perfect match.

Figure 5 plots the theoretical and numerical FEM radiation impedance for the fundamental wavenumber (12.2 rad/$\upmu$m) of gratings (a) and (b) and meander lines (c) and (d) with various number of fingers. The length of the transducers is $\ell = 10\,\upmu$m, $N = 3,\,\,5,\text{\,\,and\,\,} 9$, $a = 125$, and $\,b = 500\,$ nm. A CPW transducer of $a = 125$ nm was also simulated, and the intrinsic radiation resistance is overlayed for comparison (solid black). The film thickness was $d = 100$ nm, with a uniform out-of-plane bias field of $\hbox {H}_a = 277$ kA/m.

The agreement between theory and simulation is excellent. For a meander line, $R_r$ increases with $N^2$ due to the multiplicative effect of the array factor, $r_{af}$, while for a grating, $R_r$ always remains below the intrinsic radiation resistance, $r_i$ (solid black). As *N* increases, the transducer has a narrower response in k-space centered at 12.2 rad/$\upmu$m because the grating approximates a spatial sinusoidal function (and its harmonics). The 125 nm CPW has a lower $R_r$ than a meander with $N=3$; the CPW splits the return current into two paths of half the amplitude, so a CPW can be considered closer to an $N = 2$ meander line in terms of performance. Hence, high-order meander transducers should be chosen in place of CPW lines.

Figure 6 plots each efficiency term for the FEM simulated grating, meander, and CPW transducers. Third-order MNs with quality factors of 100 are designed using the RFT for each transducer and can be found in section Supplemental 3. A general observation is that as the number of elements increases, the radiation efficiency increases significantly for both gratings and meander lines. The CPW transducer behaves slightly worse than a meander line of $N = 3$. The number of elements does not significantly change the match efficiency since these transducers are narrow-band. As *N* increases, the dissipation efficiency improves slightly for meander lines but degrades for gratings. Since more elements in a grating reduces ohmic loss, this reduces the overall resistance of the transducer, requiring a MN with higher ITR, and resulting in more power dissipation in the MN per Eq. (3).

In principle, $\eta_r$ should increase by *N* for a grating and a meander line: $R_r$ increases by $N^2$ while $R_\ell$ increases by *N* for a meander line, whereas for a grating, $R_r$ does not change with *N* while $R_\ell$ decreases by *N*. However, Fig. 6b seems to indicate that meander lines may actually have a higher peak efficiency than gratings. Upon further investigation, the reduction in $\eta_r$ for gratings was found to be a result of distributed resistance in the feed geometry: the transfer length between each grating finger adds resistance before each finger, resulting in less current flowing through fingers furthest from the transducer input. Consequently, $R_\ell$ does not exactly follow the 1/*N* rule for parallel resistances; therefore, $\eta_r$ does not scale with *N* for a grating with the particular feed shown in Fig. 3d. A different feed that provides uniform current flow to each finger can be designed, but this is left for future work.

Figure 6c confirms that a meander line is superior in nearly every way to a grating: a meander has higher radiation efficiency for a simple feed, is easier to match to and dissipates less power in the match due to its higher radiation resistance (lower ITR), and has greater bandwidth. Thus, a meander line is more suitable than a grating for launching exchange spin waves. The only potential drawback of a meander line is that since $R_r$ increases with $N^2$ for a given current, non-linear film-behavior is reached at a lower RF input power than with a grating.

Figure 6d plots the dissipation efficiency (Eq. 3) of the MN (solid, markers) as a function of ITR for various element quality factors and is compared to Eq. (8) (dashed). The MNs were designed using the RFT and then adjusted to have finite $Q_e$. The lumped components were not optimized again for a good match, which is why the networks do not maintain a constant ITR as $Q_e$ increases. The dashed blue lines indicate the ITR required by each transducer to obtain a perfect match. As explained in “Matching considerations”, the MN for a meander line with 9 conductor elements (M-N9) incurs much less loss for a given Q-factor than a MN for a 9-element grating (G-N9) because the ITR is lower. Equation (8) is valid when $Q_e$ is much greater than the network quality factor, which is evident by the greater discrepancy between Eqs. (8) and (3) when $Q_e = 10$.

A Q-factor above 500 is considered extremely good for most passive components at microwave frequencies; transmission lines can easily have a Q-factor near 100–200. Lumped components generally have a Q-factor between 10 and 50 for frequencies below their self-resonance but are generally limited to below 6 GHz due to package parasitics and the lowest value of inductance or capacitance that can be achieved.

### Efficient, wideband multi-conductor transducers

In light of the discussion above, we briefly mention a fourth class of transducers, illustrated in Fig. 3e,f, which can provide efficient and wideband transduction by varying the dimensions of the fingers in one of two ways: chirping or fanning. Fanning the fingers provides spatially-dependent launch points, whereas chirping the fingers provides a uniform wavefront for all design frequencies and provides higher radiation resistance than the fan grating for the same overall length. While these are illustrated as gratings, they can be designed as meander lines to obtain the benefits described in the previous section. This class of transducers is conducive to filters, true-time delay lines, wave-based computing, spectrum analysis, and similar applications that require broadband performance.

## Conclusion

In this paper, we have performed a system-level analysis of spin-wave transducers using both theoretical and numerical approaches to magnetostatic-wave transducers. Our demonstrations show that transmission lines, such as microstrip and CPW, while commonly used, are not well-suited for either very high performance or flexible designs. Instead, multi-conductor meander transducers provide flexible frequency selection, bandwidth, and good efficiency even at nanoscale wavelengths. In addition, matching networks are clearly necessary to maximize transducer efficiency. However, these networks will always have to be comparable in size to an EM wavelength. This has implications for Boolean spintronics, because even if the transducer can be scaled down while maintaining high radiation efficiency, the matching network can never be scaled at a given frequency. Therefore, an alternative is to design the transducer with sufficient meanders such that its radiation impedance is close to $50\,\Omega$, thereby eliminating the need for a matching network. This allows for transducer scalability at the cost of some match efficiency. Future work should be conducted to assess how much transducers can be scaled before this approach fails to provide sufficient efficiency, such as very accurate modeling of ohmic losses and parasitics that may reduce the radiation resistance. Future work also includes experimental confirmation of these structures, simulations using non-uniform bias fields accounting for finite film dimensions, and experimental demonstrations of sub-100 nm transducers.

## Supplementary Information

Supplementary Information.
