# Supplementary material for: Efficient electromagnetic transducers for spin-wave devices
Source: Sci Rep. 2021 Sep 15;11:18378. doi: 10.1038/s41598-021-97627-3 (PMC8443574; doi:10.1038/s41598-021-97627-3)
Supplement: Supplementary file 1 — Supplementary Information. [file 41598_2021_97627_MOESM1_ESM.pdf]

# Efficient Electromagnetic Transducers for Spin-Wave Devices

**David A. Connelly[1*], Gyorgy Csaba[2], Hadrian Renaldo O. Aquino[1], Gary H. Bernstein[1], Alexei Orlov[1], Wolfgang Porod[1], and Jonathan Chisum[1*]**

[1]University of Notre Dame, Department of Electrical Engineering, Notre Dame, IN 46556, USA
[2]Pázmány Péter Catholic University, Faculty for Information Science and Bionics, Prater u. 50/a, H-1083 Budapest, Hungary
[*]dconnel7@nd.edu, jchisum@nd.edu

## Contents

**S1** Radiation Resistance Factors **2**
**S2** EM simulation setup **4**
**S3** Meander and Grating Matching **6**
**S4** Dispersion Relations **7**
References **8**

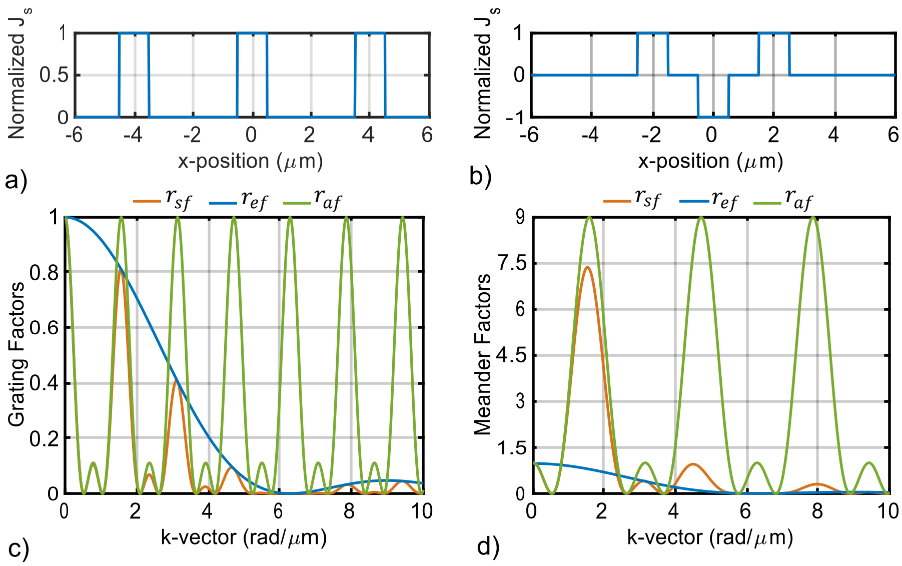


**Figure S1.** Element, array, and surface current factors for parallel and anti-parallel currents. (a) Parallel current distribution of a 3-element grating. (b) Anti-parallel current distribution of a 3-element meander line. (c) Theoretical element, array, and surface current factors for a 3-element grating (parallel currents) using Eqs. 1 and 3. (d) Theoretical element, array, and surface current factors for a 3-element meander (anti-parallel currents) using Eqs. 1 and 5. For the grating and meander, $w = 1\,\mu$m is the conductor width. For a meander, $p = 2\,\mu$m is the distance from center-to-center of each conductor. To ensure the same fundamental frequency as the meander, the grating conductors are separated by $2p$.

## S1 Radiation Resistance Factors

Figure S1(c) and (d) illustrate the difference between element ($r_{ef}$) and array ($r_{af}$) factors for three parallel (Fig. S1(a)) and anti-parallel (Fig. S1(b)) currents. For simplicity, a uniform current distribution is assumed for each element, although in reality the current is not uniform in a conductor due to skin and proximity effects. The $\mathscr{J}_{sFT}$ of a uniform current distribution (rectangle function) of a single conductor with width $w$ is the sinc function; therefore, the element factor is:

$$r_{ef} = \left|\text{sinc}\left(\frac{kw}{2}\right)\right|^2, \tag{1}$$

where $w = 1\,\mu$m is the width of each conductor in this example.

Taking the first conductor to be centered at the origin, the surface current factor for the parallel (grating) and equal-amplitude currents shown in Fig. S1(a) is

$$r_{sf} = \left|\text{sinc}\left(\frac{kw}{2}\right)\right|^2 \frac{1}{3^2}\left[1 + e^{-i2kp} + e^{-i4kp}\right]^2, \tag{2}$$

where the separation between conductors from center-to-center is $2p = 4\,\mu$m. Therefore, the array factor is

$$r_{af} = \frac{1}{3^2}\left|1 + e^{-i2kp} + e^{-i4kp}\right|^2. \tag{3}$$

Taking the first conductor to be centered at the origin, the surface current factor for the anti-parallel (meander) currents shown in Fig. S1(b) is

$$r_{sf} = \left|\text{sinc}\left(\frac{kw}{2}\right)\right|^2 \left[1 - e^{-ikp} + e^{-i2kp}\right]^2, \tag{4}$$

Therefore, the array factor for three anti-parallel and equal elements is

$$r_{af} = \left|1 - e^{-ikp} + e^{-i2kp}\right|^2. \tag{5}$$

In this example, the array factor for a meander is $3^2$, or more generally, $N^2$ for a grating with $N$ fingers because a meander guides the same current through each finger which results in an array factor that exceeds the element factor. A grating splits the incoming current into various fingers, so while it is capable of frequency selection like a meander, it does not result in the array factor exceeding the element factor. In both cases, the element factor acts as a low-pass filter. Transducers with anti-parallel currents can be designed to exceed the intrinsic and element factor response, and in turn, increase the efficiency at nanoscale wavelengths (section 4.3).

## S2 EM simulation setup

Ansys HFSS has a built-in Polder susceptibility tensor for modeling ferrites based on the small signal approximation to the Landau-Lifshitz equation. HFSS requires the input of the saturation magnetization, $M_s$, ferromagnetic resonance linewidth, $\Delta H$, the frequency at which the resonance linewidth is defined, $f_m$, and either a uniform or non-uniform effective bias field, $H_e$. Anisotropy, exchange and non-linearity are not modeled. The ferrite sample is assumed to be in saturation.

For a bias along $\hat{\mathbf{z}}$, the Polder permeability tensor, $[\bar{\mu}]$, used by HFSS is

$$[\bar{\mu}] = \mu_o\big([\bar{I}] + [\bar{\chi}]\big) = \mu_o \begin{bmatrix} 1+\chi_d & j\kappa & 0 \\ -j\kappa & 1+\chi_d & 0 \\ 0 & 0 & 1 \end{bmatrix} = \mu_o \begin{bmatrix} 1+\dfrac{\omega_o\omega_m}{\omega_o^2-\omega^2} & j\dfrac{\omega\omega_m}{\omega_o^2-\omega^2} & 0 \\ -j\dfrac{\omega\omega_m}{\omega_o^2-\omega^2} & 1+\dfrac{\omega_o\omega_m}{\omega_o^2-\omega^2} & 0 \\ 0 & 0 & 1 \end{bmatrix} \tag{6}$$

where $[\bar{\chi}]$ is the Polder susceptibility tensor, $[\bar{I}]$ is the identity matrix, $\chi_d = \frac{\omega_o\omega_m}{\omega_o^2-\omega^2}$ , $\kappa = \frac{\omega\omega_m}{\omega_o^2-\omega^2}$, $\omega = 2\pi f$, $\omega_m = \gamma\mu_o M_s$, and $\omega_o = \gamma\mu_o H_e$, $\mu_o$ is the permeability of vacuum, $\gamma$ is the gyromagnetic ratio, and $f$ is the frequency.

HFSS is not capable of modeling the exchange interaction. However, when designing transducers using closed-form expressions, the Polder susceptibility tensor can be modified easily to include the exchange field by replacing $\omega_o$ with $\omega_o^{ex} = \omega_o + \omega_m\lambda_{ex}k^2$, where $\lambda_{ex} = \frac{2A}{\mu_o M_s^2}$ is the phenomenological exchange constant, and $A$ is the exchange stiffness constant.

Designing with closed-form expressions is further advantageous because of the reduction in computational resources. For example, when simulating the $a = b = 5\,\mu$m CPW of section 4.2 using the methodology without a film outlined in section 3, a single frequency required 6 GB of RAM to solve for 104,400 tetrahedral elements in 137 seconds, whereas simulating the CPW with a film, a single frequency required 21.34 GB of RAM to solve for 234,600 tetrahedral elements in 367 seconds. The simulation computer utilizes a Intel(R) Xeon(R) Silver 4214R processor with 2x 12 cores. Simulating the CPW transducer with the film (the numerical approach) more than doubled the simulation time and mesh elements and more than tripled the memory usage because both the metal and the film have to be meshed. While this appears to be a modest saving, consider a more complicated structure, such as the $N = 9$ meander transducer of section 4.3. When simulating the meander without a film, a single frequency required 24 GB of RAM to solve for 403,800 tetrahedral elements in 613 seconds, whereas simulating the meander with a film, a single frequency required 106.8 GB of RAM to solve for 794,440 tetrahedral elements in 2,465 seconds. Simulating the meander transducer with the film (the numerical approach) more than quadrupled the simulation time and memory usage and nearly doubled the number of mesh elements. The simulation time and memory usage increased by a greater factor than the number of mesh elements increased because modeling a permeability tensor, even if for the same number of mesh elements, requires greater computational resources than modeling a constant permeability. This illustrates that the theoretical approach to designing transducers is particularly compelling when modeling more complex transducers, which are required to obtain efficient and wideband transduction.

Because HFSS does not have a built-in absorbing boundary layer (ABL) for spin waves, an ABL was manually specified on both sides of the transducer by defining a spatially-varying linewidth. Although the software requires a linewidth, the ABL function is presented here in terms of the damping parameter, $\alpha$, since it is a fundamental material property. Two different functions were utilized: an exponential function and a hyperbolic tangent function[1]. For the CPW transducers in sections 3 and 4.2, an exponential taper was utilized:

$$\alpha(y) = \alpha_{\min} e^{b|y|}, \qquad b = \frac{\ln\big(\alpha_{\max}/\alpha_{\min}\big)}{\mathrm{ABL}}, \tag{7}$$

where $\alpha_{\max} = 0.0005$, and $\alpha_{\min} = 10^{-8}$, and ABL = 100 $\mu$m is the width of the absorbing layer on either side of the transducer's center. Since $\alpha_{min}$ is so low in the film directly below the transducer, it has no noticeable effect on the impedance of the transducer.

For the meander and grating transducers in section 4.3, a hyperbolic tangent function was utilized for defining a spatially varying linewidth:

$$\alpha(y) = \Delta\alpha\left[1+\tanh\left(\frac{|Y \pm Y_{\mathrm{offset}}| - ABL}{\sigma_\alpha}\right)\right], \qquad \sigma_\alpha = \frac{-ABL}{\tanh^{-1}[(\alpha_{\min}/\Delta\alpha)-1]}, \qquad \Delta\alpha = \alpha_{\max} - \alpha_{\min}, \tag{8}$$

where $\alpha_{\max} = 0.001$, $\alpha_{\min} = 10^{-7}$, ABL $= 2.5\,\mu$m is the width of the absorbing layer on each side of the transducer, and $Y_{\mathrm{offset}}$ depends on the transducer size (number of fingers) and ensures that the ABL begins after, not directly underneath, the transducer.

These two profiles are plotted in Fig. S2.

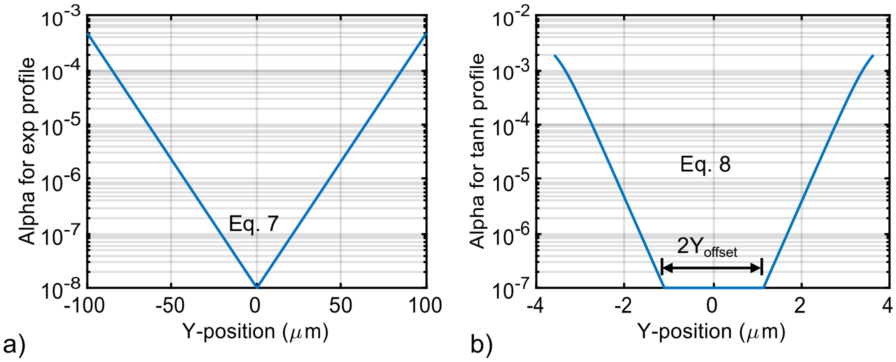


**Figure S2.** (a) Profile of damping parameter using the exponential function given in Eq. 7. (b) Profile of damping parameter using the exponential function given in Eq. 8. In this example, $Y_{\text{offset}} = 2.25\,\mu$m for the $N = 9$ meander.

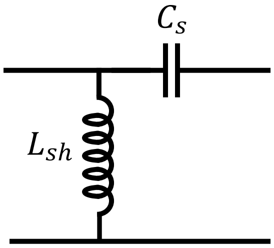


**Figure S3.** L-section MN corresponding to Tab. S1

**Table S1.** Matching Network for Meander and Gratings

| Transducer | $L_{sh}$ (pH) | $C_s$ (pF) |
|---|---|---|
| CPW 125 nm | 376 | 1.856 |
| Grating N = 3 | 179 | 3.83 |
| Grating N = 5 | 141 | 4.81 |
| Grating N = 9 | 119 | 5.76 |
| Meander N = 3 | 532 | 1.33 |
| Meander N = 5 | 747 | 0.978 |
| Meander N = 9 | 1,200 | 0.737 |

## S3 Meander and Grating Matching

Table S1 provides the shunt inductance, $L_{sh}$ and series capacitance, $C_s$, values of the L-section matching network (Fig. S3) used for the transducers of section 4.3 and Fig. 6 in the paper. The inductor and capacitor each have a quality factor of 100.

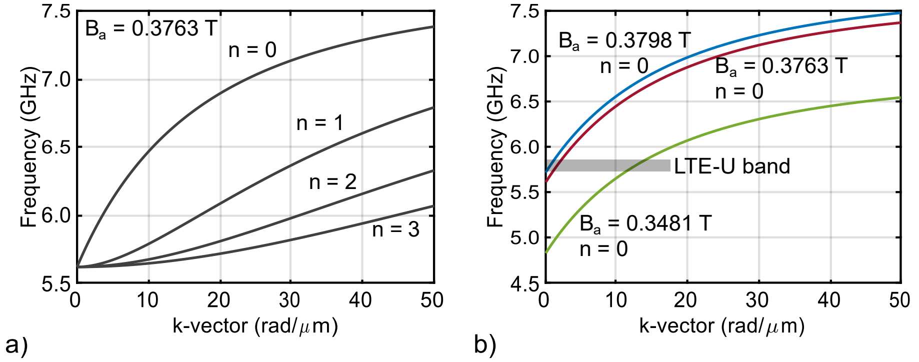


**Figure S4.** (a) First four modes of the YIG film used for the transducer designed in section 3 of the main text. (b) Fundamental mode for the transducers designed in section 4.2 and 4.3 of the main text.

## S4 Dispersion Relations

The dispersion curves for transducers modeled in sections 3, 4.2 and 4.3 of the main text are given in Fig. S4. A YIG film ($M_s = 140\,\text{kA/m}$) with thickness of 100 nm was utilized for all transducers. The exchange field was not included in these curves since these transducers are validated with HFSS, which is not able to model the exchange interaction. Figure S4(a) plots the first four thickness modes for the CPW transducer designed in section 3 of the main article with a bias of $B_a = 0.3763$ T. Because there is very little coupling to higher-order modes at low wavenumbers, only the fundamental mode (n = 0) is considered in the main text. Figure S4(b) plots the fundamental mode's dispersion relation for the $a = b = 1\,mu$m CPW of section 4.2 (red curve), the $a = b = 2,3,5\,\mu$m CPW of section 4.2 (blue curve), and the meander/grating transducers of section 4.3 (green curve). The targeted LTE-U band is highlighted in grey. The following dispersion relation is used for forward volume waves[2]

$$\tan\left[\frac{kd}{2}\sqrt{-(1+\chi_d)}-\frac{n\pi}{2}\right]=\frac{1}{\sqrt{-(1+\chi_d)}}, \tag{9}$$

where $d$ is the film thickness, $k$ is the magnitude of the in-plane wavenumber, $n$ is the thickness mode number, $\chi_d$ is the diagonal component of the susceptibility tensor and is a function of angular frequency, $\omega$.

## References


**1.** Venkat, G., Fangohr, H. & Prabhakar, A. Absorbing boundary layers for spin wave micromagnetics. *J. Magn. Magn. Mater.* **450**, 34–39, DOI: 10.1016/j.jmmm.2017.06.057 (2018).

**2.** Stancil, D. D. *Theory of Magnetostatic Waves* (Springer-Verlag, 1993).
